# Reward Network Immediate Early Gene Expression in Mood Disorders

**DOI:** 10.3389/fnbeh.2017.00077

**Published:** 2017-04-28

**Authors:** Claire E. Manning, Elizabeth S. Williams, Alfred J. Robison

**Affiliations:** Department of Physiology, Michigan State UniversityEast Lansing, MI, USA

**Keywords:** depression, reward system, immediate early gene (IEG), FosB/ΔFosB, CREB, accumbens, hippocampus, mood disorders

## Abstract

Over the past three decades, it has become clear that aberrant function of the network of interconnected brain regions responsible for reward processing and motivated behavior underlies a variety of mood disorders, including depression and anxiety. It is also clear that stress-induced changes in reward network activity underlying both normal and pathological behavior also cause changes in gene expression. Here, we attempt to define the reward circuitry and explore the known and potential contributions of activity-dependent changes in gene expression within this circuitry to stress-induced changes in behavior related to mood disorders, and contrast some of these effects with those induced by exposure to drugs of abuse. We focus on a series of immediate early genes regulated by stress within this circuitry and their connections, both well-explored and relatively novel, to circuit function and subsequent reward-related behaviors. We conclude that IEGs play a crucial role in stress-dependent remodeling of reward circuitry, and that they may serve as inroads to the molecular, cellular, and circuit-level mechanisms of mood disorder etiology and treatment.

## Introduction

Neurocircuitry has evolved to reward behaviors that contribute to evolutionary fitness with feelings of pleasure, motivating individual organisms to value, and repeat actions that increase the likelihood of propagating their genetic material. These may include having sex, eating certain foods, caring for offspring, or engaging in social activity. However, the modern human environment, replete with abundant resources and access to pleasurable stimuli, may allow increased reward processing to induce maladaptive pursuits, such as overeating or addiction to drugs or sex (Berridge and Kringelbach, 2015). Conversely, deficiency in reward processing contributes to the anhedonic symptoms of mood disorders like depression (Nestler, 2015a; Luking et al., 2016), and current treatment and research in mood disorders focuses on the circuitry underlying reward and the mechanisms that may contribute to defective reward processing.

Rewarding behaviors become favored because they are reinforced. This process requires that they: (1) give rise to positive emotions (pleasure), (2) induce learning, and (3) produce additional consummatory behavior (i.e., eating, copulating, interacting, etc.). Thus, the reward circuitry must integrate information from brain structures that drive feelings of pleasure, formation and storage of memories, and decision-making and behavioral output. It has become increasingly clear over the last two decades that changes in gene transcription within this reward circuitry contribute to the development of mood disorders (Nestler, 2015a). These disease-related changes can involve mechanisms as diverse as histone and DNA modification, non-coding RNA expression, and transcription factor induction and activity (Dalton et al., 2014; Geaghan and Cairns, 2015; Nestler, 2015a). The expression of many transcription factors involved in these processes is tightly regulated by neuronal activity, and such transcription factors belong to a class of molecules termed immediate early genes (IEGs). These IEGs represent a particularly attractive mechanism for diseases involving anhedonia, as reward circuit neuronal activity is altered in many models of depression (Russo and Nestler, 2013; Lammel et al., 2014), and thus the expression of many IEGs is dysregulated in the same models (Reul, 2014; Nestler, 2015a). Therefore, to fully unravel the etiology of human mood disorders, it is critical that we uncover the regulation of IEGs in the reward circuitry under both basal and disease conditions. This review will cover progress in identifying the regulation and downstream targets of IEGs within the brain regions comprising the reward circuitry, and the current evidence linking reward circuitry IEGs to stress responses and mood disorders.

## The cortico-basal ganglia reward network

The central feature of the reward circuitry is the release of dopamine (DA) from the ventral tegmental area (VTA) neurons into limbic brain regions that control prediction, perception, and processing of rewarding stimuli. VTA DA neurons have major projections to the prefrontal cortex (PFC; the mesocortical pathway) and to the nucleus accumbens (NAc; the mesolimbic pathway), but also project to hippocampus, amygdala, and several other forebrain regions. Mesocortical DA is thought to be involved in emotional responses and control of cognition (Nestler et al., 2015), while mesolimbic DA is traditionally linked to reward and motivated behaviors. Mesolimbic DA release activates dopamine receptors (DRs) on NAc medium spiny neurons (MSNs), GABAergic cells comprised of two largely separate populations that express predominantly either D1 or D2 DRs (Surmeier et al., 2007; Lobo, 2009). D1 MSNs comprise the “direct” pathway, which ultimately increases thalamocortical drive, while D2 MSNs make up the “indirect” pathway, which results in reduced thalamocortical drive. Because D1 DRs increase responsiveness to glutamatergic excitation while D2 DRs decrease this glutamate excitability, VTA DA release facilitates the direct pathway while putting a brake on the indirect pathway, with the combined effect of increased cortical drive.

NAc MSNs receive glutamatergic inputs from several cortical and limbic structures, including medial and lateral divisions of the PFC, ventral hippocampus (vHPC), basolateral amygdala (BLA), and medial thalamus (Sesack and Grace, 2010; Floresco, 2015). PFC inputs onto NAc regulate goal-directed behaviors, such as seeking and consuming substances/activities associated with reward, including food, sex, drugs, and social interactions (Kalivas et al., 2005; Gruber et al., 2009), providing the “executive control” required for planning and performing actions to obtain rewards. vHPC inputs onto NAc presumably provide information regarding affective valence of locations in space and previous experience generated from emotional learning. This applies to both positive and negative emotional states, i.e., reward- and aversion-based learning, including context-dependent fear conditioning, feeding behavior, and responses to drugs of abuse (Vezina et al., 1989; Fanselow, 2000; Kanoski and Grill, 2017). While general BLA activity and BLA projections to many other brain regions regulate fear-related learning and behavior, glutamatergic inputs from BLA onto NAc MSNs increases reward seeking and supports positive reinforcement (Ambroggi et al., 2008; Stuber et al., 2011; Janak and Tye, 2015).

**Figure 1:**
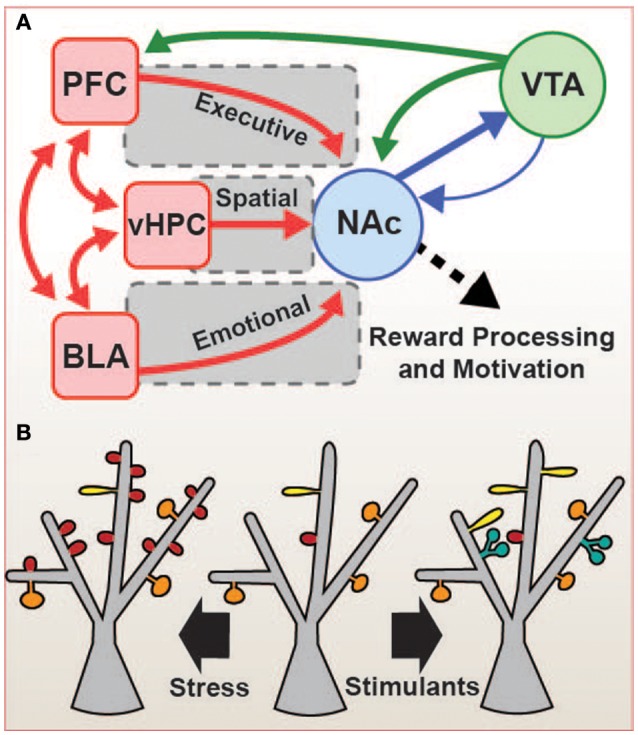
**Cortico-basal ganglia reward network. (A)** The nucleus accumbens (NAc) integrates glutamatergic inputs (red) which regulate spatial (ventral hippocampus, vHPC), emotional (basolateral amygdala, BLA), and executive (prefrontal cortex, PFC) behaviors, and these inputs are modulated by dopamine (green) from the ventral tegmental area (VTA). BLA, vHPC, and PFC are also interconnected, allowing for further integration of the circuitry. The sum of these inputs results in reward-related learning and decision-making. Stress can cause alteration in the expression of IEGs throughout these brain regions, altering the function and structure these connections, and this may result in pathological changes in reward perception and motivation, including the anhedonia or despair common to many mood disorders. **(B)** Strength and number of glutamatergic connections correlate with changes in the shape and number of dendritic spines in NAc. In chronic social defeat stress (CSDS, left), an increase in the number of stubby (red) spines is observed, while in stimulant drug administration (e.g., cocaine and amphetamines, right), the numbers of thin (yellow), and branched (blue) spines are increased. These structural changes may be mediated by IEGs, and may represent a key factor in the circuit-level changes observed in the depressed and addicted disease states.

Many of these NAc glutamatergic input regions also project to each other, and NAc MSNs send and receive GABAergic projections to and from the VTA as well. This results in a complex cortico-basal ganglia reward network (Sesack and Grace, 2010; Floresco, 2015), a simplified version of which is presented here (Figure 1A). The ultimate function of this network is to regulate and integrate cortical/limbic glutamatergic signals representing executive control, memory, and emotion with dopaminergic reward processing to control the thalamocortical outputs that drive behavior. Critically, many of the regions involved in this circuit undergo long-term changes in gene expression, and cell function, often as a result of stress exposure, that may drive mood-related disorders, and these changes result, in part, from aberrant expression and function of IEGs. This is particularly evident in stress-induced changes in the structure of reward network neurons.

Chronic social defeat stress, a rodent model of depression, causes an increase in dendritic spine density in NAc MSNs (Figure 1B). MSN dendritic spines are the structural correlate of glutamatergic inputs, and the number and shape of spines represent the number and strength of those individual inputs. The increased spine density observed in the NAc after chronic social defeat stress (CSDS) is due primarily to an increase in the number of stubby spines, which are immature, and there is no change in mature mushroom-shaped spines (Christoffel et al., 2011). Stubby spines are associated with smaller postsynaptic densities (PSDs) and weaker responses to glutamate, but the increase in their density after stress may represent an increase in glutamatergic signaling to the NAc, and it is indeed accompanied by an increase in the number (but not amplitude) of miniature excitatory postsynaptic potentials (mEPSPs; Christoffel et al., 2011). In addition to stress paradigms such as CSDS, the administration of psychostimulants such as cocaine also increases dendritic spine density, mainly due to an increase in the number of thin spines (Robinson and Kolb, 1999; Russo et al., 2010), a shape also considered immature. However, in contrast to stress, stimulant drug administration increases dendritic spine complexity in NAc MSNs, with many spines showing branching with multiple heads (Robinson and Kolb, 1999; Figure 1B). This increase in complexity may represent a reorganization and increase in synaptic signaling, indicating a change in circuit function after drug experience. Many gene products may be involved in the regulation of dendritic spines in the stressed and drug-exposed states, including several of the IEGs discussed below (e.g., ΔFosB, CREB; Maze et al., 2010; Russo et al., 2010). A better understanding of the links between IEG expression and structural and functional plasticity of the reward network is critical to the development of our understanding of mood and addiction pathologies.

## cAMP response element-binding protein (CREB)

**Figure 2:**
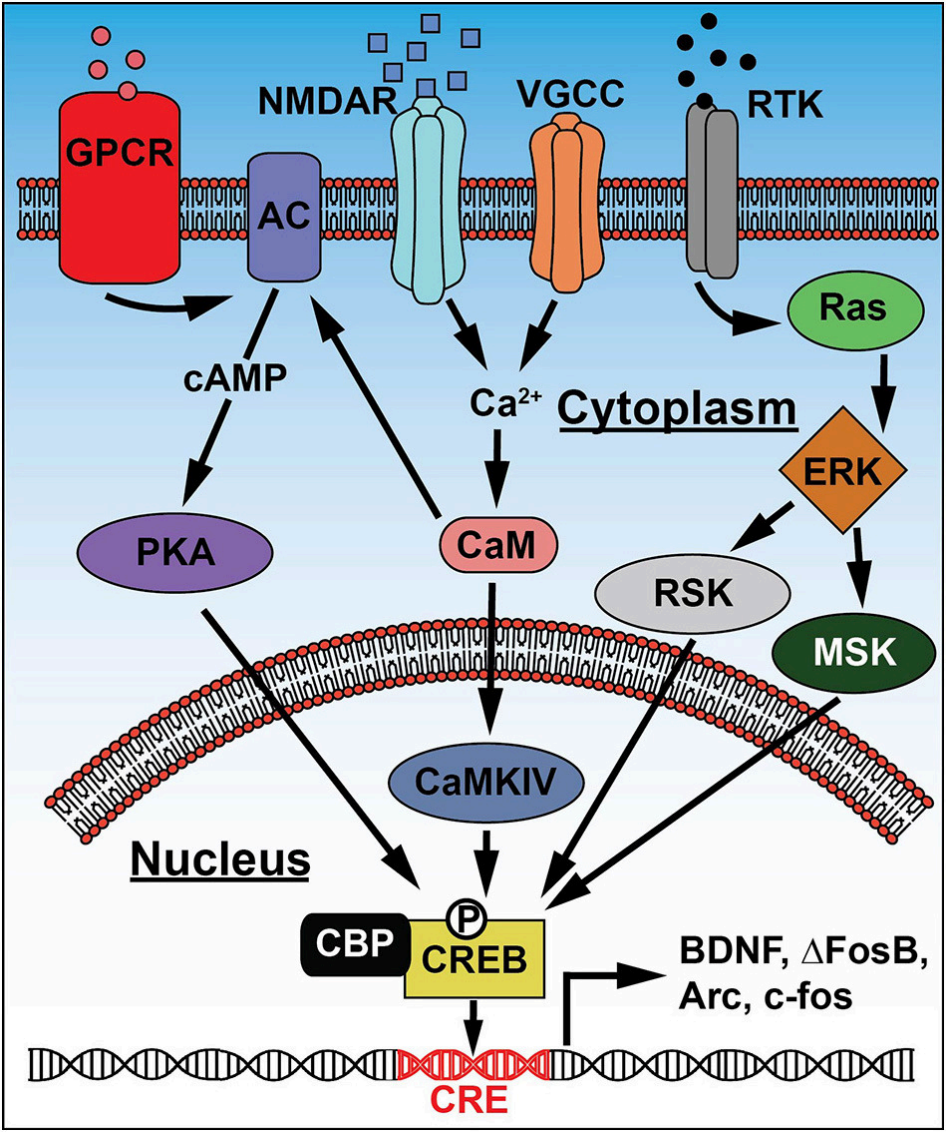
**Signaling pathways leading to CREB activation**. Extracellular signals and changes in membrane potential activate receptors and channels including: G-protein coupled receptors (GPCR), NMDA-type glutamate receptors (NMDAR), voltage gated Ca^2+^ channels (VGCC), and receptor tyrosine kinases (RTK). These generate increases in second-messenger (cAMP and Ca^2+^) or MAPK signaling that converge on kinase activation: protein kinase A (PKA), Ca^2+^/calmodulin-dependent protein kinase IV (CaMKIV), extracellular signal-regulated kinase (ERK), ribosomal S6 kinase (RSK), and mitogen and stress-activated kinase (MSK). Subsequent phosphorylation at Ser133 activates CREB and promotes interaction with CREB-binding protein (CBP), causing CREB to bind cAMP response elements (CRE) in the promoter regions of target genes and increase expression of proteins that regulate neuronal function, like BDNF and IEGs such as Arc, ΔFosB, and c-fos.

CREB is a transcription factor that binds to the canonical cAMP response element (CRE) in DNA in response to activation of signaling pathways involving cAMP, Ca^2+^/calmodulin, or various growth factors and/or cytokines. CREB activation of target gene transcription (Figure 2) is controlled by phosphorylation at serine 133 by protein kinase A (PKA, downstream of cAMP), Ca^2+^/calmodulin-dependent protein kinase IV (CaMKIV, downstream of Ca^2+^), and/or MAP kinase signaling (downstream of growth factors and cytokines; Kida and Serita, 2014). Ser133 phosphorylation promotes interaction with CREB-binding protein (CBP), a critical step for transcriptional activation (Chrivia et al., 1993). The earliest and most extensive studies of CREB's role in neuronal function centered on its control of gene transcription underlying long-term potentiation of synapses and memory formation. CREB is critical for memory and synaptic plasticity in the invertebrate sea slug *Aplysia* (Dash et al., 1990; Kandel, 2012) and fruit fly (Yin et al., 1994), and long-term memory is impaired in CREB loss-of-function mice, but enhanced in CREB gain-of-function mice, primarily due to its role in the hippocampus (summarized in Kida and Serita, 2014).

CREB is stimulated in NAc by exposure to various stressors, and its activation in NAc has been linked to a variety of emotional responses, with the general consensus being that chronic activation of CREB in NAc leads to anhedonia while inhibition of CREB function in NAc promotes reward (Barrot et al., 2002; Carlezon et al., 2005). Moreover, reduced CREB activity in NAc appears to have antidepressant-like effects in multiple stress models (Pliakas et al., 2001; Conti et al., 2002; Newton et al., 2002; Covington et al., 2011), suggesting that stress-induced CREB activation in NAc may contribute to the etiology of depression. However, the opposite appears true with regard to anxiety-like behaviors, as increased NAc CREB activity appears anxiolytic while inhibition of NAc CREB promotes anxiety (Barrot et al., 2002, 2005; Wallace et al., 2009), indicating that manipulation of NAc CREB activity may not be a simple therapeutic inroad to treatment of mood disorders.

In contrast to its function in NAc, CREB activation in hippocampus produces an antidepressant effect (Chen et al., 2001), and it is indeed induced in the hippocampus by a variety of antidepressant treatments (Nibuya et al., 1996; Thome et al., 2000). One of the many identified target genes of CREB is brain-derived neurotrophic factor (BDNF), and BDNF is also induced in hippocampus by antidepressants (Nibuya et al., 1995) and it is a key transducer of antidepressant effects (Björkholm and Monteggia, 2016). This CREB-BDNF pathway has been postulated to induce hippocampal neurogenesis as a crucial step in antidepressant action (Duman, 2004; Carlezon et al., 2005). It therefore follows that dysfunction of CREB in hippocampus may underlie both depression and some of the cognitive dysfunction linked to chronic stress that are often comorbid with mood disorders (Bortolato et al., 2014). It is also critical to note that CREB regulates the expression of many other IEGs linked to stress responses and depression, including FosB, c-fos, and Arc (see below), and so may act as a master regulator of the activity-dependent transcriptional response to stress throughout the reward circuitry.

## AP-1 proteins—c-fos, FosB/ΔFosB, Jun

Activator protein 1 (AP1) is a complex composed of heterodimers between Fos family proteins, Jun family proteins, Jun dimerization proteins, and/or activating transcription factor (ATF) proteins that, when assembled, act as potent and specific regulators of gene transcription. A typical AP1 complex consists of Fos-Jun heterodimers that utilize leucine zippers present in both proteins for dimerization and a basic region that interacts with DNA. The Fos family of transcription factors is comprised of c-fos, FosB (and its splice variants, ΔFosB and Δ2ΔFosB), Fra1, and Fra2, all of which are induced by neuronal activity. c-fos is transiently and robustly induced, with a half-life ranging from minutes up to a couple hours (Sheng and Greenberg, 1990; Kovács, 1998; Ferrara et al., 2003), and is hypothesized to target a wide variety of genes associated with cell differentiation, cell and synapse development, synaptic plasticity, and learning (Alberini, 2009; West and Greenberg, 2011). Its clear connection to cellular activity has led to its use as a marker of brain region activation in a range of behavioral and physiological conditions, however conclusive evidence for c-fos-specific gene targets has not yet been provided, and its direct role in neuronal function remains obscure. It is induced throughout the reward circuitry by virtually all emotionally salient stimuli (Kovács, 1998; Cruz et al., 2015; Nestler, 2015b), but its functional role in mood disorders and antidepressant responses is not well-understood.

FosB is encoded by the *FosB* gene and shares many characteristics with c-fos: FosB has low basal expression and is transiently and robustly induced by neuronal activity (Nestler et al., 1999), with a similar short half-life in cells to that of c-fos (Dobrazanski et al., 1991; Ferrara et al., 2003; Ulery et al., 2006). Splice variation of *FosB* gene transcripts produces a premature stop codon resulting in the truncated ΔFosB protein, which lacks two c-terminal degron domains lending it increased stability (Carle et al., 2007). Most other IEGs have a half-life of a few hours, while ΔFosB has an unusually long-half life, up to 7 days *in vivo* (Hope et al., 1994; Andersson et al., 2003; Ulery-Reynolds et al., 2009), making it a marker of *chronic* neuronal activity. ΔFosB is induced throughout the reward circuitry by chronic stress (Perrotti et al., 2004) and chronic antidepressant exposure (Vialou et al., 2015), and like CREB (which is essential for ΔFosB induction, Vialou et al., 2012), the behavioral effects of its expression differ by brain region. In the NAc, ΔFosB is induced by chronic social defeat stress, and its induction is greater in animals resilient to the behavioral effects of stress than in those susceptible to the depression-like phenotype (Vialou et al., 2010a). Moreover, ΔFosB induction in NAc promotes resilience to chronic stress and is necessary for the antidepressant effects of SSRIs like fluoxetine (Vialou et al., 2010a), apparently through modulation of AMPA receptor subunit expression and epigenetic regulation of CaMKIIα expression (Vialou et al., 2010a; Robison et al., 2014). Its induction by stress in resilient mice appears specific to D1-type MSNs in NAc, while a lower level of induction is seen in D2-type MSNs of susceptible mice (Lobo et al., 2013). Indeed, the specific overexpression of ΔFosB in D1 MSNs appears to have antidepressant effects (Vialou et al., 2010a; Muschamp et al., 2012; Donahue et al., 2014), and it alters the structure of glutamatergic synapses on these specific neurons. ΔFosB promotes the expression of immature thin and stubby dendritic spines, and a concomitant increase in silent synapses, in D1 but not D2 MSNs (Grueter et al., 2013), suggesting that it selectively alters glutamatergic inputs onto NAc direct pathway output neurons, directly modulating reward processing.

In the medial PFC, ΔFosB is selectively induced in mice susceptible to chronic social defeat stress (Vialou et al., 2014). Further, in direct opposition to its effects in NAc D1 MSNs, ΔFosB inhibition in mPFC neurons promotes resilience to chronic stress, while ΔFosB overexpression drives susceptibility, at least in part through induction of the cholecystokinin-B receptor (Vialou et al., 2014). The effect appears to be mediated by ΔFosB expression in mPFC neurons that project to NAc, emphasizing the critical nature of activity-dependent gene expression within the circuitry of reward. We recently reported that ΔFosB expression in hippocampus is critical for multiple forms of learning (Eagle et al., 2015), but the role of hippocampal ΔFosB in stress responses and mood disorders, both locally and in projections to NAc or PFC, remains unknown.

## Serum response factor (SRF)

SRF is a transcription factor that binds specifically to the serum response element found in the promoters of many other IEGs and a number of cardiac-specific genes (Knöll and Nordheim, 2009). In the adult brain, SRF is required for activity-induced gene expression and synaptic plasticity but not for neuronal survival (Ramanan et al., 2005). Through its mediation of the expression and function of cytoskeletal-associated proteins, SRF seems to be instrumental in converting synaptic activity into plasticity-associated structural changes in neuronal circuits (Knöll and Nordheim, 2009), making it a potential player in the activity-dependent gene expression underlying stress-induced changes in reward circuitry. Indeed, SRF is induced in the NAc of resilient mice after chronic social defeat stress, and it binds to the *FosB* promoter and increases transcription of the gene (Vialou et al., 2010b). The subsequent SRF-dependent stress induction of ΔFosB is critical for the resilient phenotype, and, unlike cocaine-dependent induction of ΔFosB, appears independent of CREB actions at the *FosB* promoter (Vialou et al., 2010b, 2012).

## Early growth response protein-1 (Egr-1)

Egr-1, also known as zinc finger protein 268, is an activity-dependent neuronal transcription factor that binds DNA via three distinct zinc finger domains. It appears to play a role in neuronal plasticity (Knapska and Kaczmarek, 2004), perhaps through its regulation of the expression of synaptobrevin II (Petersohn and Thiel, 1996). Egr-1 is induced in hippocampus by acute stress, like forced swim in rats, through activation of a complex epigenetic mechanism stemming from hippocampal glucocorticoid receptor (GR) activation (summarized in Reul, 2014). MAPK signaling downstream of GRs drives MSK1 and Elk-1 activity, a pathway also upstream of CREB and c-fos induction. This favors Ser10 phosphorylation and Lys14 acetylation of histone 3 at the Erg-1 gene promoter, leading to relaxed chromatin compaction, changes in DNA methylation, and Erg-1 expression (Gutièrrez-Mecinas et al., 2011; Saunderson et al., 2016). This effect lasts at least days in the brain, and may be responsible for subsequent altered responses to forced swim, perhaps underlying long-term stress-induced despair, a hallmark of mood disorders. Indeed, Egr-1 expression is reduced in both hippocampus and PFC by social isolation (Ieraci et al., 2016), indicating that it may contribute to long-term changes in mood due to prolonged stress. In the future, it will be critical to determine whether the effects of Egr-1 expression in hippocampus occur due to alteration of hippocampal projections to or from other reward circuitry components, such as NAc.

Egr-3, which colocalizes with Egr-1 and is also induced in an activity-dependent manner, has recently been implicated in multiple mood disorders. Egr-3's many targets include Arc (Li et al., 2005), discussed below, as well as NMDA and GABA receptor subunits (Roberts et al., 2005; Kim et al., 2012), suggesting that it may contribute to excitatory/inhibitory balance in reward circuitry. Initial studies using SNPs in the Egr-3 gene found a potential association with child bipolar disorder (Gallitano et al., 2012). A more recent study used large-scale microarray data and found that Erg-3 may play a critical role in dysregulation of PFC transcriptional networks in patients with bipolar depression (Pfaffenseller et al., 2016). Moreover, rodent studies suggest that Egr-3 may underlie some of the effects of clozapine in treating both psychosis and bipolar symptoms (Gallitano-Mendel et al., 2008; Williams et al., 2012), suggesting that further study of Egr-3 may yield critical insights into the etiology of mood disorders.

## NPAS4

Neuronal PAS domain protein 4, or NPAS4, is an activity-dependent transcription factor expressed exclusively in neurons. It is necessary for normal development of inhibitory interneurons as well as neuronal plasticity in response to experience (Lin et al., 2008; Ploski et al., 2011; Ramamoorthi et al., 2011; Sim et al., 2013). Since NPAS4 is induced in both excitatory and inhibitory neurons and initiates distinct cascades in each cell type (Spiegel et al., 2014), it is thought to regulate excitatory and inhibitory balance within circuits (Bloodgood et al., 2013). Identified downstream targets of NPAS4 include brain-derived neurotrophic factor (BDNF) in excitatory neurons, and FERM and PDZ domain-containing protein 3 (Frmpd3) in inhibitory neurons (Spiegel et al., 2014).

In HPC, NPAS4 induction by both synaptic potentiation and depression protocols requires MAPK and PI3K pathways (Coba et al., 2008), suggesting a link to activation of other IEGs, like CREB. Stress directly mediates NPAS4 activation, as agonist bound glucocorticoid receptor binds to the NPAS4 promoter to downregulate its expression during acute stress (Furukawa-Hibi et al., 2012). After chronic stress, NPAS4 mRNA is significantly decreased in the hippocampus of juvenile mice, and these NPAS4-deficient juveniles developed cognitive deficits in adulthood (Ibi et al., 2008; Yun et al., 2010; Coutellier et al., 2015). These long-term changes may arise through epigenetic regulation, as the NPAS4 promoter has several CpG islands, and stress increases methylation at these sites (Furukawa-Hibi et al., 2015). Several animal strains, including SERT knockout rats and the Flinders Sensitive Line, have shown correlations between low NPAS4 expression, depressive-like behaviors, and antidepressant resistance (Guidotti et al., 2012; Bigio et al., 2016). Much of this work has been done in HPC, and further studies are needed to characterize the role of NPAS4 in NAc and other reward circuitry areas in the context of the same depression models. Moreover, NPAS4 is upregulated in NAc after exposure to drugs of abuse (Guo et al., 2012), but is role in drug responses or behaviors underlying addiction remains unknown.

## Activity-regulated cytoskeleton-associated protein (Arc)

Arc is a flexible, modular, multidomain protein that interacts with many partners (Myrum et al., 2015; Zhang et al., 2015). Through these interactions, Arc serves to maintain the phosphorylation of the actin depolymerization factor cofilin, preserving its inactive form, and thus favors the polymerization of actin (Messaoudi et al., 2007). In this manner, Arc promotes the induction of thin, immature dendritic spines, a function shared with ΔFosB (see above). Importantly, Arc is also localized to the postsynaptic density where it plays a critical role in internalization of AMPA receptors (Chowdhury et al., 2006) and promotes formation of immature dendritic spines (Peebles et al., 2010) and long-term depression (LTD; Bramham et al., 2010).

Recent evidence suggests that Arc expression and function may be tied to multiple aspects of depression. In a variety of rat and mouse paradigms, Arc is consistently induced throughout the cortex and hippocampus by acute stress, but may be up- or down-regulated by chronic stressors depending on the paradigm (Elizalde et al., 2010; Molteni et al., 2010; Boulle et al., 2014). In addition, the vast majority of studies suggest that chronic antidepressant treatment induces Arc expression throughout rodent cortex and hippocampus, and stress-induced Arc expression in specific brain regions appears to predict the subsequent effects of stress on cognitive function (summarized in Li et al., 2015). Thus, it seems possible that stress- or antidepressant-induced Arc may be critical for remodeling of reward circuitry synapses, perhaps in glutamatergic inputs to NAc or connections between other cortical and basal-ganglia regions, but further study will be required to determine the exact contribution of Arc expression to stress responses and mood disorders.

## Homer1a

Homer1 proteins act primarily as scaffolds mediating the interactions and locations of other neuronal proteins, including metabotropic glutamate receptors (e.g., mGluR1 and mGluR5), IP_3_ receptors, Shank, and others. The short splice variant of Homer1, Homer1a, is induced by neuronal activity and acts as a dominant negative to block interactions of the long, constitutively active splice variants (Homer1b and Homer1c) with their normal ligands via competition for EVH1 binding sites. For example, Homer1a has been shown to uncouple mGluR receptors from downstream signaling (Tu et al., 1998) as well as cause a decrease in the size and density of dendritic spines (Sala et al., 2003) via inhibition of Shank targeting to synapses. The *Homer1* gene is implicated in the pathogenesis of major depression through genome-wide association as well as neuroimaging studies (Rietschel et al., 2010). In a repeated forced swim mouse model of depression, Homer1a is reduced in cortex, and this is reversed by antidepressant exposure (Sun et al., 2011). Interestingly, Homer1b and 1c are induced in HPC by social defeat stress (Wagner et al., 2015), and increasing their levels in proportion to Homer1a may act as a mechanism of resilience. This is because overexpression of Homer1a in mouse HPC promotes susceptibility to social defeat stress, with overexpressing animals showing increased behavioral despair and less active coping behavior (Wagner et al., 2015). In the accumbens, Homer1a is induced by antipsychotics that act at dopamine receptors (reviewed in Iasevoli et al., 2009), but any role of Homer1a in accumbens-mediated behavioral responses to stress and drugs of abuse remains to be uncovered.

## Outstanding questions and future directions

Despite accumulating evidence of IEG induction within reward circuitry in rodent models and patients with mood disorders, we still don't fully understand the contribution of IEGs to reward circuitry function and pathological behavior. A critical next step is to target IEGs in specific neural circuits. Such an approach has been difficult using classical techniques, but recent advances in cell labeling and cell- and circuit-specific manipulation provide exciting avenues to address some critical outstanding questions.

### Are there distinct roles for IEGs in specific neuronal subtypes?

Do IEGs perform the same functions in all neuronal cell types? Because some IEGs are induced more sparsely compared to others (e.g., NPAS4), the relevance of IEG expression to mood disorders may be tied to their induction in specific cell populations. Transgenic mouse lines allowing selective overexpression or knockout of IEGs in neurons that produce specific neurotransmitters (i.e., DAT-Cre or GAD-Cre) or express specific receptors (i.e., D1-Cre or D2-Cre) will be a critical tool in future studies. Moreover, coupling these lines with Cre-dependent viral vectors will address the role of IEGs in individual neuronal subtypes with both spatial and temporal specificity.

### What is the role of IEGs in specific brain circuits?

**Figure 3:**
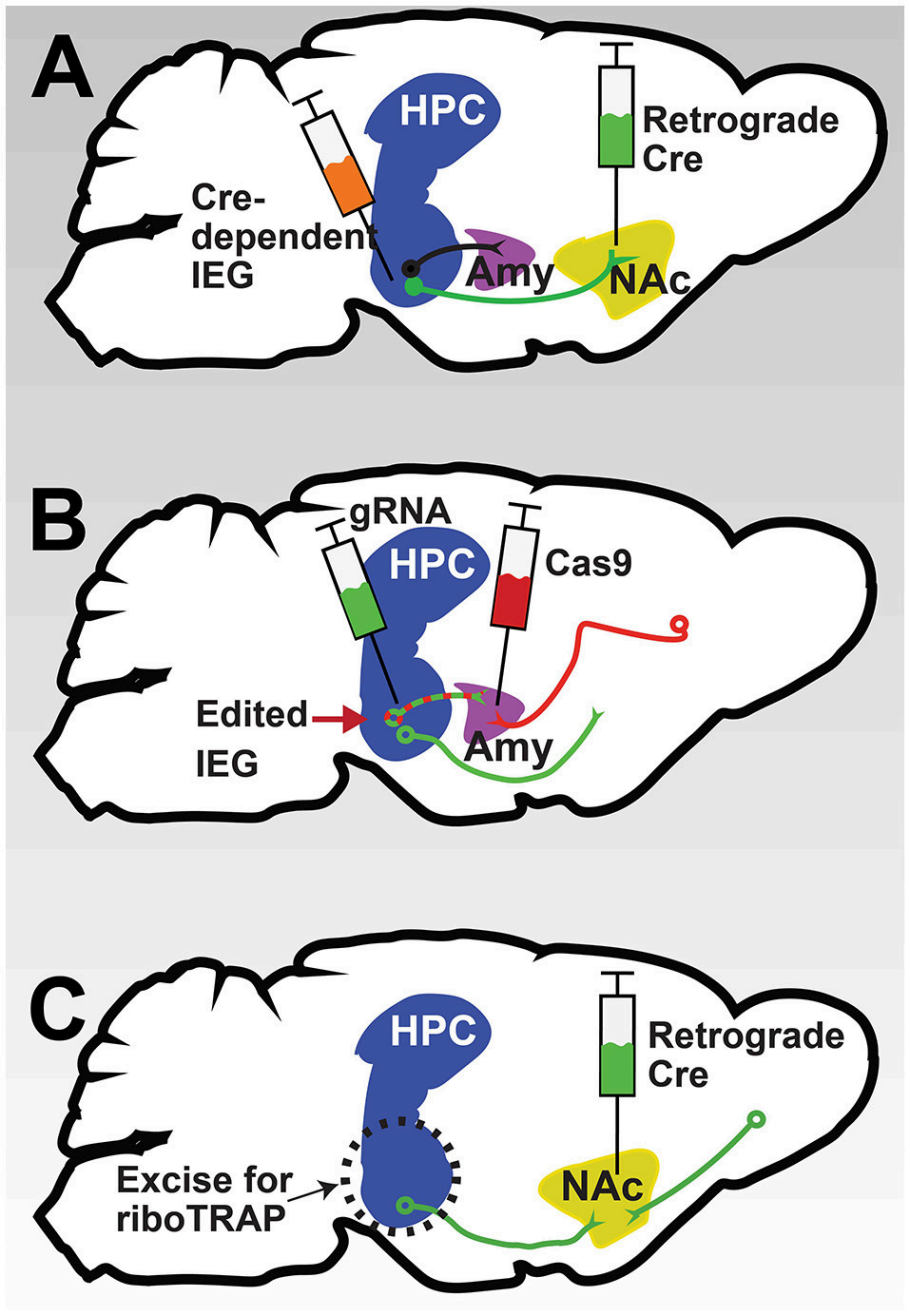
**Potential methods for circuit-specific interrogation of IEG function. (A)** Schematic depicting the combination of a retrograde Cre virus (green) injected into a target region like NAc with a local virus expressing an IEG in a Cre-dependent manner injected into a projecting region like ventral HPC. Such a strategy would result in overexpression of the IEG only in HPC neurons projecting to NAc. **(B)** Schematic of a converse strategy: the combination of a retrograde virus expressing Cas9 (red) in a target region like amygdala (Amy) with a local virus expressing a guide RNA targeting an IEG (green) in a projecting region like ventral HPC could be used to silence an IEG in a specific circuit. **(C)** Schematic of a strategy for uncovering circuit-specific gene expression. Using a retrograde Cre virus in a target region to induce expression of GFP-tagged ribosomes in the projection region allows the use of TRAP to determine gene expression changes in the circuit. By combining this approach with mice floxed for a specific IEG, circuit-specific IEG transcriptional targets could be revealed.

Although IEGs may be activated in many brain regions in response to stress or drugs, their relevance in circuits underlying addiction and depression behaviors is not fully understood. To assess the contribution of activated IEG suites within mesolimbic and cortical circuitry to cell function and animal behaviors, novel retrograde viral vector approaches will be critical. For instance, by combining a retrograde virus expressing Cre injected into a target region such as NAc with a locally expressing virus overexpressing an IEG of interest in a Cre-dependent manner injected into ventral HPC, one could measure the effects of the IEG on the function of HPC neurons specifically projecting to NAc, as well as subsequent behavior of the animal (Figure 3A). Alternatively, by combining retrograde expression of the Cas9 enzyme with a local expression of guide RNA, CRISPR-mediated editing of an IEG could be used to determine its circuit specific role (Figure 3B), an approach currently being piloted by our group and others. Of course, these techniques could be combined with transgenic Cre driver lines described above to allow cell-type *and* circuit-specific manipulation of IEGs, critical steps for our understanding of their role in the pathophysiology of psychiatric disease.

### What are the gene targets of IEGs in specific cell types and circuits?

Although it is critical to understand the roles of IEGs in specific cell types, neuronal ensembles, and specific circuits, many IEGs make unlikely pharmacological targets for treatment of psychiatric disease, as they often play critical roles in non-disease-related brain regions and other tissues. However, uncovering the gene targets of IEG transcription factors, like Fos family proteins or NPAS4, may reveal critical mediators of pathophysiology that are more amenable to pharmacological manipulation. New advances in gene expression profiling, like translating ribosomal affinity purification (TRAP; Heiman et al., 2014), are sufficiently flexible and robust to be applied to Cre-dependent cell- and circuit-specific approaches described above (Lobo, 2009; McCullough et al., 2016), and primed for use in Cre-dependent ensemble-specific approaches (Sakurai et al., 2016). Utilizing Cre-dependent reporter mouse lines expressing GFP-tagged ribosomes in combination with retrograde Cre viruses will allow circuit-specific TRAP profiling of gene expression (Figure 3C). Combining such an approach with mice floxed for a specific IEG will then allow assessment of the contribution of that IEG to circuit-specific gene expression in the context of stress or drugs. We predict that such techniques will uncover novel gene products underlying mood or substance use disorders that could be pharmacologically accessible targets for novel treatments.

## Conclusions

It is clear that exposure to stressful events in life increases risk for mood disorders, and the many preclinical and fewer postmortem studies summarized here suggest that this may arise in part from stress-induced remodeling of reward circuitry driven by IEG expression. For some of these IEGs, like CREB, Homer1a, and ΔFosB, evidence abounds for their roles in stress responses, multiple aspects of mood disorders, drug addiction, and even antidepressant treatment, and the challenge now lies in integrating their functions across the brain regions and cell types involved and determining their downstream targets in order to uncover potential novel drug targets. For other IEGs, such as Egr-1, NPAS4, and Arc, their induction by stress makes them molecules of interest in mood disorder research, but causal connections to depression-related behaviors have not yet been uncovered, and continued study of their role in reward circuitry function is needed. In all cases, it has become clear that stress-dependent remodeling of the reward circuitry, and particularly of glutamatergic inputs to NAc, is a critical component in the development of depression- and addiction-related phenotypes, and that IEGs play a crucial role in this process and may provide a pathway to the molecular, cellular, and circuit-level mechanisms of mood disorder etiology and treatment.

## Author contributions

CM, EW, and AR researched, wrote, and edited the manuscript.

## Funding

This work was funded by awards to AR from the National Institute of Mental Health (1R01MH111604-01) and the Whitehall Foundation (2013-08-43).

### Conflict of interest statement

The authors declare that the research was conducted in the absence of any commercial or financial relationships that could be construed as a potential conflict of interest.
